# Intravenous Penicillin for Antenatal Syphilotherapy

**DOI:** 10.1155/S1064744993000031

**Published:** 1993

**Authors:** Henry L. Galan, Paul M. Yandell, Alfred B. Knight

**Affiliations:** Department of Obstetrics and Gynecology Texas A&M University Health Science Center Scott and White Clinic and Memorial Hospital, 2401 S. 31st St. Temple TX 76508 USA

## Abstract

A 21 year old woman (G_2_ P_0101_) of 24 weeks gestation presented with syphilis of unknown duration. Sonography revealed fetal hydrops and placental thickening. Weekly intramuscular injections of 2.4 million U Bicillin for 3 weeks was initiated as recommended by the Centers for Disease Control. Repeat sonogram 1 week after starting treatment revealed increased ascites and a new pericardial effusion. Due to the worsening fetal condition, therapy was altered and the patient was admitted for IV penicillin. She received a continuous infusion of 18 million U penicillin G daily for 10 days. Serial sonograms showed improvement offetal ascites and pericardial effusion with 10 days of IV therapy, and complete resolution of hydrops was noted within 3 weeks. The fetus was born at term with no stigmata of congenital syphilis on newborn exam, and laboratory tests suggested adequate treatment in utero.


Infectious Diseases in Obstetrics and Gynecology 1:7–11 (1993)
© 1993 Wiley-Liss, Inc.

### Intravenous Penicillin for Antenatal Syphilotherapy

**Henry L. Galan, Paul M. Yandell, and Alfred B. Knight**
*Department of Obstetrics and Gynecology, Texas A&M University Health Science Center, Scott and White Clinic and Memorial Hospital, Temple, TX*

### Abstract

**A 21 year old woman ($G_2\ P_{0101}$) of 24 weeks gestation presented with syphilis of unknown duration. Sonography revealed fetal hydrops and placental thickening. Weekly intramuscular injections of 2.4 million U Bicillin for 3 weeks was initiated as recommended by the Centers for Disease Control. Repeat sonogram 1 week after starting treatment revealed increased ascites and a new pericardial effusion. Due to the worsening fetal condition, therapy was altered and the patient was admitted for IV penicillin. She received a continuous infusion of 18 million U penicillin G daily for 10 days. Serial sonograms showed improvement of fetal ascites and pericardial effusion with 10 days of IV therapy, and complete resolution of hydrops was noted within 3 weeks. The fetus was born at term with no stigmata of congenital syphilis on newborn exam, and laboratory tests suggested adequate treatment in utero.** © 1993 Wiley-Liss, Inc.

Key words
Fetal hydrops, congenital syphilis, *T. pallidum*

In 1987 the incidence of syphilis reached its highest peak since 1950. This increase was greater in women than men, regardless of ethnic group.[1] Furthermore, the increase in congenital syphilis paralleled the rise in women.[2] Benzathine penicillin G is considered the treatment of choice for maternal infection by the Centers for Disease Control (CDC); it prevents neonatal infection in 97–100% of cases.[3–5] However, if there is reinfection, severe infection, or treatment late in pregnancy (second or third trimester), treatment failure can occur.[5,6] The CDC has reported that these failures are responsible for 35% of neonatal infections.[2,7] In the past 20 years, no prospective, randomized study evaluating fetal syphilotherapy has been published.[5,7]

Left untreated, congenital syphilis has significant perinatal morbidity and mortality, with reported mortality rates of 40–54%.[1,8,9] Treatment failure in severe fetal infection may result in fetal death.[3,5] In a recent case report, Hallak et al. attempted to treat a hydropic fetus with high-dose IV penicillin.[10] They were unable to complete therapy due to fetal distress during fetal blood transfusion that resulted in an emergent cesarean section. We describe a pregnancy with a severely affected fetus that had complete resolution of fetal hydrops and placental thickening 3 weeks after initiation of maternal IV infusion of penicillin G with no clinical stigmata of congenital syphilis identified at birth.

### CASE REPORT

A 21 year old black woman ($G_2P_{0101}$) at 24 weeks gestation by menstrual and ultrasound dating was diagnosed with syphilis of unknown duration at her initial prenatal visit with the local health department. There was no prior history of syphilis. Her obstetrical history was complicated by prior preterm delivery at 36 weeks gestation in which serology testing for syphilis was negative. She had had multiple sexual contacts in the past. Her rapid plasma reagin (RPR) was 1:64 and microhemag-

Address correspondence/reprint requests to Dr. Alfred B. Knight, Dept. of Ob/Gyn, Scott & White Clinic, 2401 S. 31st St., Temple, TX 76508.

Received June 10, 1992
Accepted December 19, 1992

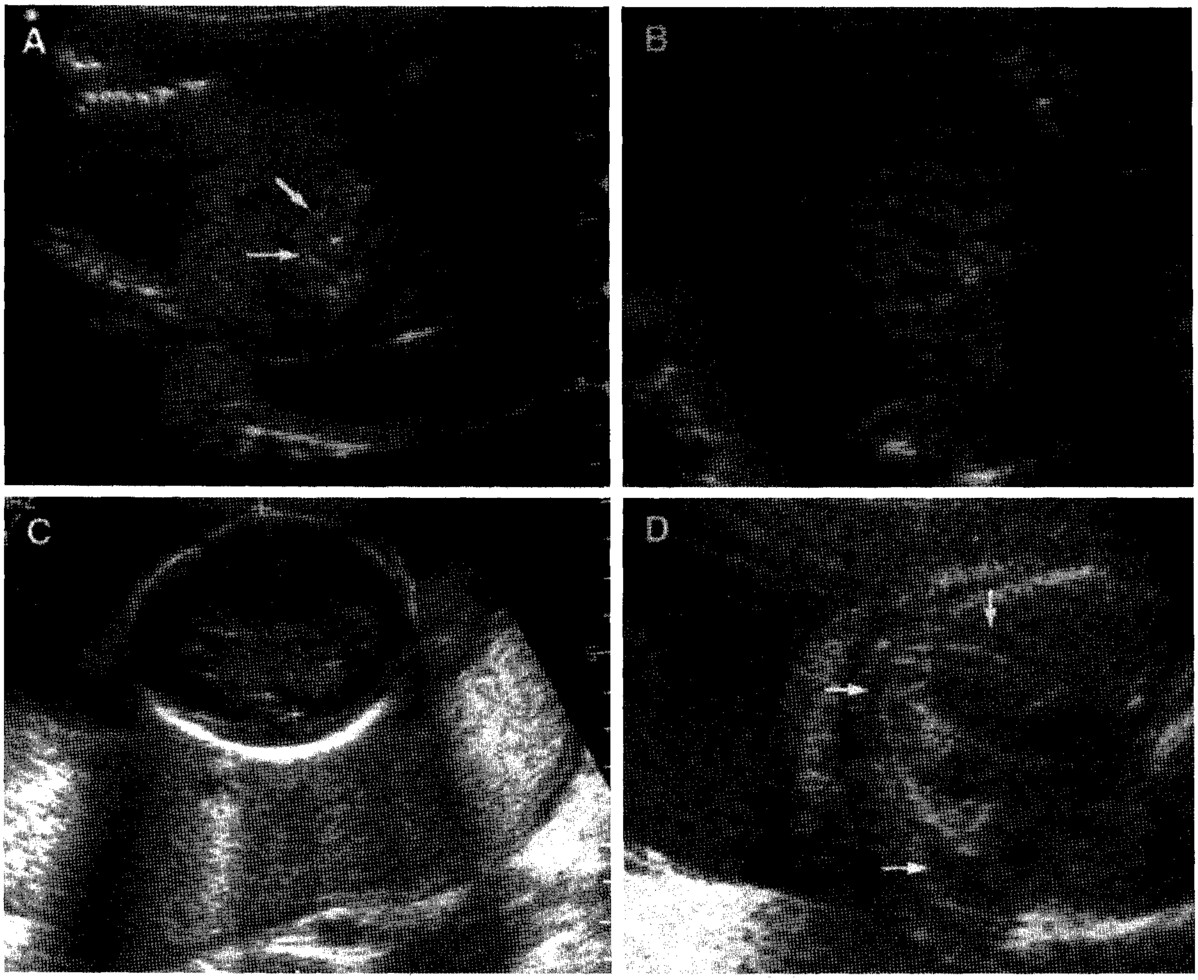

Fig. 1. Fetal sonograms prior to high-dose intravenous penicillin. **A:** Disproportion of fetal abdomen to thorax. Echodensities in the bowel (arrows). Ascites noted in periphery of abdomen. **B:** Dilated loops of small bowel within marked ascites. **C:** Thickened placenta measuring 5–7 cm. **D:** Pericardial effusion (arrows).

glutination assay for antibodies to *Treponema pallidum* (MHATP) was reactive. Spinal tap was not performed because of a normal neurologic examination. She was started on weekly injections of 2.4 million U of Bicillin for 3 weeks as recommended by the CDC for syphilis of unknown duration. An ultrasound performed at the start of therapy revealed fetal hydrops and placental thickening, an enlarged liver, intestinal echodensities, and dilated loops of small bowel. Parvovirus B-19 titers showed immunity. A repeat ultrasound 8 days after initiation of therapy revealed a pericardial effusion and progression of the ascites (Fig. 1).

Due to worsening fetal conditions despite 1 week of Bicillin treatment, the patient was admitted to the antepartum service for aggressive IV penicillin G therapy with doses routinely used to treat adult neurosyphilis. Daily penicillin G at 18 million U continuous IV infusion was initiated and continued for 10 days. The patient tolerated treatment well and remained afebrile throughout her hospitalization. She did not experience the Jarisch-Herxheimer reaction other than a few uterine contractions requiring no tocolysis. No fetal heart rate abnormalities were noted during episodic monitoring.

Serial ultrasound was performed throughout the hospitalization. After 4 days of therapy the ascites and pericardial effusion were unchanged. Six days after therapy there was slight improvement of the ascites. On day 8 of therapy significant improve-

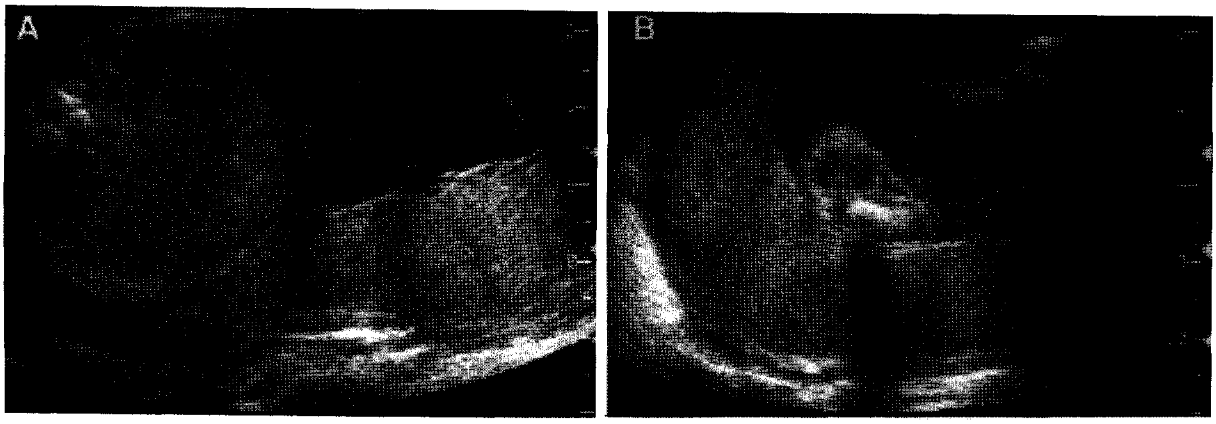

**Fig. 2. Fetal sonograms 3 weeks following initiation of high-dose intravenous penicillin. A: Resolution of ascites. B: Normal-appearing placenta.**

ment of both ascites and pericardial effusion were noted. On the day of discharge, following 10 days of IV penicillin, ultrasound revealed even less ascites and the pericardial effusion was just detectable. Three weeks after initiation of treatment, follow-up ultrasound revealed complete resolution of fetal hydrops and placental thickening (Fig. 2). There was good interval growth, and the placenta was no longer hydropic. Ultrasound was performed at 2–4 week intervals until delivery, and all remained normal.

The patient delivered at 37+ weeks gestation after the onset of spontaneous labor. No stigmata of congenital syphilis were evident on the newborn examination. Complete blood count showed no evidence of anemia or thrombocytopenia. Liver function tests were normal. Umbilical cord Venereal Disease Research Laboratory (VDRL) titer was 1:2. Fluorescent treponemal antibody-absorption (FTA-ABS) on serum was 3+ reactive. Cerebral spinal fluid (CSF) VDRL was non-reactive, FTA-ABS was 3+ reactive, and FTA-ABS IgM was negative. CSF gram stain and cultures were negative. The CSF protein was elevated at 194 mg/dl (normal 15–60), glucose was 37 mg/dl (normal 50–80), and the cell count was unremarkable. At the 4 week check, the infant was doing well and had surpassed its birth weight. Repeat VDRL was weakly reactive with 0 dilutions. Repeat CSF studies showed an improved, yet persistantly elevated protein of 123 mg/dl. At the 2 month check, the infant was progressing well and was neurologically intact. VDRL was nonreactive and FTA-ABS was 3+ reactive. It was felt that the infant had been treated adequately in utero and that the decreasing levels of CSF protein represented a normalizing trend. However, because the consequences of early childhood neurosyphilis are tragic and there was concern about the ability to follow this child closely over a longer period of time, the infectious disease consult service chose to treat the child again with 200,000 U of aqueous penicillin G IM twice daily for 14 days.

### DISCUSSION

With the decline in incidence of Rh isoimmunization, fetal hydrops is more commonly of infectious origin.[11] A review of the literature by Hallak et al. revealed only 14 cases of nonimmune hydrops due to syphilis. Other infectious etiologies of nonimmune hydrops sited were cytomegalovirus, bacteria, toxoplasmosis, rubella, herpes, *Listeria,* and *Chlamydia.*[10] Although fetal hydrops due to syphilis is rare, it is likely to increase along with the increasing incidence of congenital syphilis.

The current CDC recommended treatment for syphilis in the pregnant individual is similar to that of the nonpregnant individual.[9] The literature reflects uncertainty regarding the efficacy of this treatment, as shown by the number of studies reporting treatment failures, which account for 35% of neonatal infections.[2,5,6,12,13] A well-controlled evaluation of the current CDC-recommended treatment for syphilis in pregnancy has not been reported, in part because of our inability to measure the response of the disease to therapy.[3,5,6]

However, sonography is a mechanism by which the response to therapy of the severely infected fetus can be monitored.

In a 1988 publication, Wendel notes that women treated in the late second or third trimester of pregnancy may deliver affected infants prematurely or experience fetal demise soon after therapy, the latter possibly occurring secondary to the Jarisch-Herxheimer reaction. He also states that benzathine penicillin may prevent congenital syphilis, but that it may not alter the course of severe fetal disease. Several reports have documented high perinatal mortality with fetal hydrops due to syphilis.[14–17] The hydropic fetus has a poor prognosis in spite of having a treatable infection, hence the decision for aggressive parenteral therapy of the mother.

Newborns with congenital syphilis can present with overwhelming, multisystem infection or be entirely asymptomatic, with the only diagnostic test a positive serology. The severely infected syphilitic fetus demonstrates hepatosplenomegaly, skin and scalp edema, ascites, pleural effusion and fibrosis, cardiac effusion, gastrointestinal adhesions, and hematopoietic changes.[18] Ultrasound can detect and monitor placental thickening, hepatomegaly, ascites, hydrops, and small bowel dilatation.[14,19–22] Cordocentesis has been used in a small number of cases. These infected fetuses demonstrate anemia, thrombocytopenia, and elevated liver enzymes.[21]

A recent study by Wendel et al. described a hydropic fetus treated by current CDC recommendations that had resolution of hydrops 4 weeks after treatment. This fetus had mild hepatosplenomegaly at birth and required additional treatment in the neonatal period.[21] We describe placental thickening and progressive fetal hydrops that showed sonographic evidence of improvement during high-dose IV penicillin treatment with subsequent complete resolution of hydrops 3 weeks after initiation of therapy. The neonate was asymptomatic and had biochemical evidence of treated syphilis infection.

Penicillin G carries a category B risk factor and is commonly used in obstetrical infections. Prior to 1950, adverse fetal outcome due to maternal penicillin G administration was reported, but was likely due to impurities of the preparation. No evidence of birth defects induced by penicillin G has been documented. It passes rapidly into the fetal circulation and amniotic fluid in therapeutic levels, except in the first trimester, when levels may be subtherapeutic in the amniotic fluid. Parenteral penicillin G reaches equal levels in maternal serum and amniotic fluid within 60–90 minutes.[23] Therefore, doses that are normally used to treat adult neurosyphilis may attain serum and amniotic fluid levels high enough to treat severe infections in utero.

The rapid response to high-dose penicillin G therapy and lack of syphilis stigmata at birth suggest that this means of treatment may serve a role in the severely affected fetus, particularly late in pregnancy. The availability of high-resolution sonography provides a means to closely monitor the response of the disease to treatment closely . Further investigation is warranted to evaluate the efficacy of high-dose IV penicillin versus current CDC recommended treatments for the severely syphilitic fetus.

### REFERENCES

1. Centers for Disease Control: Syphilis and congenital syphilis, United States, 1985–1988. MMWR 37:486–489, 1988.
2. Centers for Disease Control: Congenital syphilis, United States, 1983–1985. MMWR 35:625–628, 1986.
3. Thompson SW: Treatment of syphilis in pregnancy. J Am Vener Dis Assoc 3:159–167, 1976.
4. Nelson NA, Struve VR: Prevention of congenital syphilis by treatment of syphilis in pregnancy. JAMA 161:869–872, 1956.
5. Wendel GD: Gestational and congenital syphilis. Clin Perinatol 15:287–303, 1988.
6. Mamunes P, Cave UG, Budell JW, et al.: Early diagnosis of neonatal syphilis: Evaluation of a gamma M-fluorescent treponemal antibody test. Am J Dis Child 120: 17–21, 1970.
7. Centers for Disease Control: Guidelines for the prevention and control of congenital syphilis. MMWR 37:s-1, 1988.
8. Hira SK, Bhat GJ, Patel JB, et al.: Early congenital syphilis: Clinicoradiographic features in 202 patients. Sex Transm Dis 12:177–183, 1985.
9. Chapel TA: Congenital syphilis. Compr Ther 14:25–28, 1988.
10. Hallak M, Peipert JF, Ludomirsky A, Byers J: Nonimmune hydrops fetalis and fetal congenital syphilis—a case report. J Reprod Med 37:173–176, 1992.
11. Watson J, Campbell S: Antenatal evaluation and management in nonimmune hydrops fetalis. Obstet Gynecol 67: 589–592, 1986.
12. Zenker PN, Rolfs RT: Treatment of syphilis. Rev Infect Dis 12(6):s590–603, 1989.
13. Ricci JM, Fojaco RM, O'Sullivan MJ: Congenital syphilis: The University of Miami/Jackson Memorial Medical Center experience, 1986–1988. Obstet Gynecol 74: 687–693, 1989.
14. Hill LM, Maloney JB: An unusual constellation of sonographic findings associated with congenital syphilis. Obstet Gynecol 78:895–897, 1991.
15. Chawla V, Pandit PB, Nkrumah FK: Congenital syphilis in the newborn. Arch Dis Child 63:1393–1394, 1988.
16. Chiang W, Wei P: Immunoglobulins in hydrops fetalis. Am J Obstet Gynecol 114:816–818, 1972.
17. Bulova SI, Schwartz E, Harrer WV: Hydrops fetalis and congenital syphilis. Pediatrics 42:285–287, 1972.
18. Ingall D, Musher D: Syphilis. In: Infectious Diseases of the Fetus and Newborn infant. Klein JO, Remington JS, ed., Saunders, Philadelphia, pp 335–374, 1991.
19. Lucas MJ, Theriot SK, Wendel GD: Doppler diastolic and systolic ratios in pregnancies complicated by syphilis. Obstet Gynecol 77:217–222, 1991.
20. Yiu-Chiu V, Chiu L: Sonographic features of placental complications in pregnancy. AJR 138:879–885, 1982.
21. Wendel GD Jr, Sanchez PJ, Peters MT, Harstad TW, Potter LL, Norgard MV: Identification of *Treponema pallidum* in amniotic fluid and fetal blood from pregnancies complicated by congenital syphilis. Obstet Gynecol 78:890–894, 1991.
22. Satin AJ, Twickler DM, Wendel GD: Congenital syphilis associated with dilation of fetal small bowel. J Ultrasound Med 11:49–52, 1992.
23. Briggs GG, Freeman RK, Yaffe SJ: Drugs in Pregnancy and Lactation. Williams and Wilkins, Baltimore, pp 482–485, 1990.
